# Patterns of inflammation and the use of reversibility testing in smokers with airway complaints

**DOI:** 10.1186/1471-2466-6-11

**Published:** 2006-06-01

**Authors:** Niels H Chavannes, Juanita HJ Vernooy, Tjard RJ Schermer, Jan A Jacobs, Mieke A Dentener, Chris van Weel, Onno CP van Schayck, Emiel FM Wouters

**Affiliations:** 1 Department of General Practice, Caphri Research Institute, Maastricht University, The Netherlands; 2 Department of Respiratory Medicine, University Hospital Maastricht, The Netherlands; 3 Department of General Practice/Family Medicine, Radboud University Nijmegen MC, The Netherlands; 4 Department of Medical Microbiology, University Hospital Maastricht, The Netherlands

## Abstract

**Background:**

Although both smoking and respiratory complaints are very common, tools to improve diagnostic accuracy are scarce in primary care. This study aimed to reveal what inflammatory patterns prevail in clinically established diagnosis groups, and what factors are associated with eosinophilia.

**Method:**

Induced sputum and blood plasma of 59 primary care patients with COPD (n = 17), asthma (n = 11), chronic bronchitis (CB, n = 14) and smokers with no respiratory complaints ('healthy smokers', n = 17) were collected, as well as lung function, smoking history and clinical work-up. Patterns of inflammatory markers per clinical diagnosis and factors associated with eosinophilia were analyzed by multiple regression analyses, the differences expressed in odds ratios (OR) with 95% confidence intervals.

**Results:**

Multivariately, COPD was significantly associated with raised plasma-LBP (OR 1.2 [1.04–1.37]) and sTNF-R55 in sputum (OR 1.01 [1.001–1.01]), while HS expressed significantly lowered plasma-LBP (OR 0.8 [0.72–0.95]). Asthma was characterized by higher sputum eosinophilic counts (OR 1.3 [1.05–1.54]), while CB showed a significantly higher proportion of sputum lymphocytic counts (OR 1.5 [1.12–1.9]). Sputum eosinophilia was significantly associated with reversibility after adjusting for smoking, lung function, age, gender and allergy.

**Conclusion:**

Patterns of inflammatory markers in a panel of blood plasma and sputum cells and mediators were discernable in clinical diagnosis groups of respiratory disease. COPD and so-called healthy smokers showed consistent opposite associations with plasma LBP, while chronic bronchitics showed relatively predominant lymphocytic inflammation compared to other diagnosis groups. Only sputum eosinophilia remained significantly associated with reversibility across the spectrum of respiratory disease in smokers with airway complaints.

## Background

Smoking causes chronic airway complaints in a significant proportion of the population [1]. The primary care physician and respiratory nurse specialist are generally the first to assess patients with a chronic cough, complaints of dyspnea or sputum production [2]. Ideally, common causes like COPD and asthma are differentiated by spirometry with reversibility testing, performed and interpreted by sufficiently trained staff [3,4].

Sputum induction is a valid and noninvasive method to assess airway inflammation, which is Patients with COPD [5,6]There is evidence of different cellular and cytokine activation in induced sputum from smoking compared with nonsmoking asthmatics; a positive relationship is apparent between smoking history, neutrophilic airway inflammation and deteriorating lung function in smoking asthmatics [7]. In mild asthmatics, but also in other respiratory patients, sputum, bronchoalveolar lavage and blood measure different compartments of inflammation [8]. The proportion of eosinophils in sputum is a more accurate marker of asthmatic airway inflammation than the proportions of blood eosinophils [9], suggesting a predominantly local systemic inflammatory process of lung tissue.

Patients with COP show significantly higher percentages of neutrophils and levels of soluble TNF receptor p55 (sTNF-R55) and the chemokine interleukin IL-8 in sputum as compared with control subjects [10]. Both neutrophils and eosinophils seem to be activated by IL-8 [11,12], which seems to be closely related with the degree of airflow obstruction [13]. In the circulation, increased levels of inflammatory markers such as lipopolysaccharide binding protein (LBP) and sTNF-R55 have been reported [14].

We studied the relation between patterns of inflammation and clinical diagnosis in primary care patients. By careful history taking (symptoms, smoking behaviour, atopic history) and measuring lung function including reversibility in smoking patients with respiratory complaints, we distinguished four clinical diagnosis groups: COPD (according to GOLD [1]), asthma, chronic bronchitis (GOLD 0) and a control group of 'healthy smokers' (no respiratory symptoms, no obstruction). Aim of the study was not to change or confirm these clinical diagnoses, but to reveal any specific inflammatory patterns underlying disease groups. We also tested what factors were associated with eosinophilia, a possible target for treatment.

## Methods

### Patient selection

The COOPT-study (COPD On Primary Care Treatment) recruited a heterogeneous group of (current or past) smokers with mild to moderate COPD and chronic bronchitis from 46 general practices in the Netherlands [15]. Reversibility was not an exclusion criterion, but any history of atopy, eczema or asthma was. Patients with an FEV_1 _between 40–88% of predicted values were included; mean post-BD FEV_1 _was 66% of predicted value (ranging from GOLD 0 to GOLD 3). A subgroup of the COOPT-population underwent sputum induction at the lung function laboratory of University Hospital Maastricht; only those in whom sputum induction was successful were included in analyses. In addition, we recruited smoking control patients with asthma features (atopy, eczema, previous recurrent episodes of wheezing, breathlessness, chest tightness, and/or cough and variable airflow obstruction to inhaled salbutamol) and smokers without respiratory complaints ('healthy smokers') from general practices not taking part in the COOPT-study, who subsequently also underwent sputum induction. Respiratory symptoms, smoking behaviour, atopic features, careful clinical history taking, and lung function measurements including reversibility determined the diagnostic category into which subjects were classified, analogue to earlier studies [10,14]. Patients with COPD thus had an absence of atopic features, but a smoking history of at least 15 packyears and a persistent obstruction, while patients with asthma had an atopic history and a variable obstruction, with a recorded reversibility >10%. Chronic bronchitics had chronic respiratory complaints for at least 3 months during the past two years but no obstruction, while 'healthy smokers' had a considerable smoking history but no chronic respiratory complaints.

During the recruiting phase, considerable effort was put into age matching, since age has been shown to affect sputum induction results [15]. Exclusion criterion was any inhaled or oral corticosteroid use in past six weeks, since this is known to possibly influence sputum induction results and underlying systemic inflammatory patterns [18,19]. Furthermore, increased respiratory complaints or signs of respiratory tract infection during four weeks preceding the study was considered a criterion for exclusion. The control subjects lived in the same geographical area as the patient population. The study protocol was approved by the Medical Ethics Committee at the University Hospital Maastricht, and written informed consent was obtained from all subjects.

### Pulmonary function testing and blood and sputum sampling

Trained lung function technicians measured FEV_1 _before and 15 minutes after inhalation of ß-agonist (salbutamol 400 ug) via a metered-dose inhaler, using a spirometer (Masterlab; Jaeger, Würzburg, Germany), according to ATS-criteria [2]. Before sputum induction (8:00 to 10:00 A.M.) took place, blood samples were collected in evacuated blood collecting tubes containing ethylenediaminetetraacetic acid (EDTA; Sherwood Medical, St. Louis, MO). Plasma samples were stored at -80°C until analyzed. Sputum was induced according to a procedure reported elsewhere [12]. Briefly, subjects inhaled 3% hypertonic saline, nebulized via an ultrasonic nebulizer (NEB2000; TEFA-Portanje, Woerden, The Netherlands) during three 7-minute periods. To minimize saliva contamination, subjects were instructed to mouthwash thoroughly with saline solution before expectoration. The collected sputum was pooled and kept at 4°C for not more than 2 hours prior to further processing. The volume of the pooled sputum (without selection of sputum plugs) was recorded, and an equal volume of 0.2% dithiothreitol (DTT; Sputolysin; Calbiochem, La Jolla, CA) was added. The samples were then mixed gently by a vortex mixer and incubated for 20 minutes at room temperature to ensure complete homogenization. Cell-free supernatants were frozen at -80°C until subsequent analysis. The total cell count and cell viability were assessed using a standard hemocytometer (Coulter Z1; Coulter Electronics, Mijdrecht, The Netherlands) and by trypan blue exclusion, respectively. Cytospins were stained according to the May-Grünwald-Giemsa method. An observer who was blinded to the clinical characteristics carried out the differential cell counts, counting 500 nucleated cells. The numbers of squamous cells were substracted, and the differential cell counts were expressed as corrected percentages. A sample was considered adequate if the slides contained 15% or less squamous epithelial cells.

### Measurements of inflammatory mediators in sputum and plasma

sTNF-R were measured in sputum supernatant and plasma using specific sandwich enzyme-linked immunosorbent assay (ELISA) as described earlier [20,21], which were not affected by presence of TNF-α [22], indicating measurement of total amounts of sTNF-R55 and sTNF-R75 (free and bound to TNF-α). Total TNF-α (free TNF-α and TNF-α bound to sTNF-R) was determined using a commercially available ELISA (HyCult Biotechnology BV, Uden, The Netherlands). IL-8 and LBP levels were determined using specific sandwich ELISA as described previously [19,23,24]. The lower detection limit was 60pg/ml for sTNF-R55, 30pg/ml for sTNF-R75, 20pg/ml for total TNF-α, 8 pg/ml for IL-8 and 1ng/ml for LBP. The presence of DTT resulted in less than 5% inhibition of the detection of sTNF-R55, IL-8 and LBP and less than 10% inhibition in case of sTNF-R75 and total TNF-a fraction, indicating that DTT has little or no effect on the assays used in this study (data not shown).

### Statistical analysis

Results are presented as mean ± SD for normally distributed variables and median (range) otherwise. Crossectional data analysis was performed on the four diagnostic categories: COPD, asthma, chronic bronchitis and 'healthy smokers'. Cell patterns and inflammatory mediators in induced sputum were analysed, and compared with inflammatory mediators in blood samples. Cut-off point for sputum eosinophilia was ≥ 3% [22]. Patterns of inflammatory markers per diagnosis and clinical factors associated with eosinophilia were analyzed in an adjusted backward regression model, the differences expressed in odds ratios (OR) with 95% confidence intervals. A p value of less than 0.05 denotes the presence of a significant statistical difference. (Statistical Package for the Social Sciences, version 11.0 for Windows; SPSS Inc., Chicago, IL).

## Results

Table 1 shows the population characteristics, indicating moderate obstruction (average FEV_1 _post BD 59% predicted; GOLD 2) and highest level of packyears in patients with COPD, while asthmatics have fewer packyears and clinically relevant reversibility. Healthy smokers and chronic bronchitis (GOLD 0) patients express normal lung function despite considerable packyears, while females are overrepresented among healthy smokers, especially compared to COPD. No significant differences with regard to age appear between diagnosis groups.

In table 2 the inflammatory patterns in different diagnosis groups are presented. Univariately, COPD was associated with sputum neutrophilia (OR 1.09 [1.04–1.15]), elevated IL-8 (OR 1.4 [1.1–1.7]), sTNF-R55 (OR 1.005 [1.0–1.01]) and sTNF-R75 (OR 1.005 [1.0–1.01]) in sputum, and raised levels of IL-8 (OR 2.5 [1.01–6.0]), sTNF-R55 (OR 7.6 [1.6–35.1]), sTNF-R75 (OR 10.2 [2.2–47.8]) and LBP (OR 1.1 [1.04–1.25]) in plasma,. Asthma patients showed a positive association with sputum eosinophils (OR 1.3 [1.05–1.5]), and inversely with sputum neutrophils OR 0.96 [0.9–0.99]), while patients with chronic bronchitis (GOLD 0) showed raised levels of lymphocytes (OR 1.5 [1.1–1.9]) in sputum, and significantly lower levels of sTNF-R55 (OR 0.1 [0.02–0.7]) and sTNF-R-75 in plasma (OR 0.1 [0.2–0.8]). Healthy smokers showed a lowered LBP (OR 0.8 [0.7–0.95]) in plasma, but no other significant relations. In table 3 these (positive or negative) univariate patterns of inflammation are indicated by + and -, while ++ and – indicate multivariate patterns of (positive or negative) significant relations.

### Multivariate associations

Multivariately, COPD remained significantly associated with sTNF-R55 in sputum (OR 1.01 [1.001–1.01]) and raised plasma-LBP (OR 1.2 [1.04–1.37]), while HS expressed significantly lowered plasma-LBP (OR 0.8 [0.72–0.95]) by contrast (see Table 3). Asthma was characterized by higher sputum eosinophilic counts (OR 1.3 [1.05–1.54]), while CB (GOLD 0) showed a significantly higher proportion of lymphocytic counts in sputum (OR 1.5 [1.12–1.9]).

**Figure 1:**
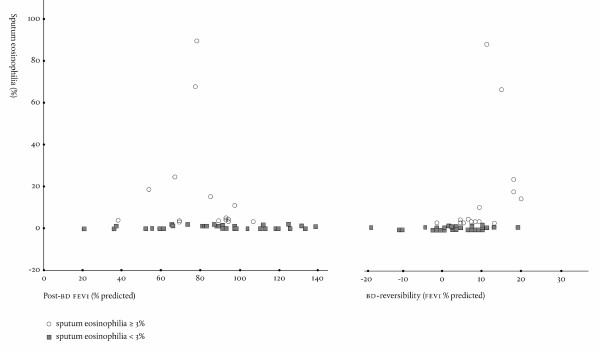
Sputum eosinophilia (≥ 3%) occurs in a wide range of patients with airway obstruction (1A) and is associated with bronchodilator reversibility (1B).

Clinically, sputum eosinophilia (≥ 3%) [22] was the only variable significantly associated with reversibility (OR 1.2 [1.04–1.36]) after adjusting for smoking, lung function, age, gender and allergy in the backward regression model. Figure 1 illustrates that sputum eosinophilia occurs across the spectrum of respiratory disease, independent from degree of obstruction; post-bronchodilator FEV_1_s are between 4–0110% of predicted. The independent relationship between reversibility and sputum eosinophilia was somewhat stronger when choosing the more recently proposed higher cut-off point of 4.6% [14]: OR 1.3 [1.0–81.53].

## Discussion

In this study we demonstrate that distinct inflammatory patterns in sputum and blood are discernable in clinical diagnosis groups of respiratory disease in smokers with airway complaints. Univariately, COPD was associated with sputum neutrophilia, and raised levels of IL-8, sTNF R55 and sTNF R75 in both sputum and plasma, and an increased level of plasma LBP. By contrast, chronic bronchitics (GOLD 0) were associated with raised lymphocytes and lowered sTNF R55 and sTNF R75 plasma-levels. 'Healthy smokers' showed a lowered plasma-LBP level, while asthma patients showed a pattern of lowered neutrophils but high eosinophils. Multivariately, COPD remained associated with raised sputum-sTNF R55 and plasma-LBP, in contrast with a significantly and independently lowered plasma-LBP in 'healthy smokers'. In asthma patients, only the high level of sputum eosinophilic inflammation remained independently associated, which underlines the difference between the sputum and plasma compartments in differentiating disease. Chronic bronchitics (GOLD 0) retained a relative predominance of lymphocytic inflammation in plasma compared to the other clinical diagnosis groups, which could point at a subpopulation of T cells in this phenotype. The considerably raised sputum-sTNF R55 and R75 in COPD-patients is in line with earlier studies of inflammation [10,14], pointing at structural changes in the airway wall as a result of prolonged and ongoing smoking-induced inflammation. Interestingly, the high levels of plasma-LBP but not in sputum of COPD-patients suggests a blood born systemic component, which may open a possibility for disease differentiation. By contrast, the lowered plasma-LBP in 'healthy smokers' is a new finding that warrants further investigation, possibly pointing at genetic differences in the inflammatory cascade. The clear distinction in inflammatory patterns between 'at risk' (GOLD 0) and COPD patients is an intriguing finding, which confirms earlier findings from the longitudinal Copenhagen City Heart Study, in which a gradual development from GOLD 0 to GOLD 1 or higher levels of obstructive disease could not be demonstrated [23]. It now seems justified on the basis of inflammatory patterns to make a clear distiction between chronic bronchitis (GOLD 0) and 'true COPD' (i.e. GOLD 1,2,3 and 4).

Furthermore, eosinophilia occurs across the spectrum of clinical diagnosis groups, remaining associated with reversibility, in contrast with all other clinical variables. It seems that the recent ATS/ERS Standards for the diagnosis and treatment of COPD recommendation to 'treat patients showing a clinically relevant reversibility as asthmatics' is in line with these findings [2]. Fabbri and coworkers [14] found that despite fixed airflow obstruction similar to COPD, a history of asthmatic symptoms remains closely related to eosinophilic inflammation. Indeed, they concluded that different inflammatory pathways do exist in patients with fixed airway obstruction. Airway inflammation with eosinophils has been reported to occur not only in asthma but also in other airway diseases such as chronic cough, allergic rhinitis and COPD [27]. Sputum eosinophilia has been associated with an accelerated decline in FEV1 and the development of COPD [28]. Induced sputum analysis allows detection of sputum eosinophilia in clinical practice [29], which is clinically important since it may be linked to corticosteroid response in COPD [22,30,31]. By contrast, there is increasing evidence that an absence of sputum eosinophilia is associated with steroid resistance [32,33]. The proportion of eosinophilic COPD seems to be limited, and of debatable clinical relevance: 4 out of 17 (24%) COPD-diagnoses when using the ≥ 3% cut-off for sputum eosinophilia, but only 1 out of 17 (6%) when using the more recently proposed cut-off of 4.6% [14]. In our chronic bronchitis group, we found only 1 out of 14 (7%) when using the cut-off of 4.6%, while the group of 'healthy smokers' expressed no eosinophilia.

Through multivariate analysis confounding differences in baseline were corrected for, and insight was achieved into the relative importance of each variable; most previous diagnostic studies have not used such statistical techniques. Furthermore, careful age matching during the recruitment phase resulted in sufficiently comparable diagnosis groups, since age is known to affect sputum induction results, especially neutrophilia [15]. Based on the same reasoning, we excluded the use of inhaled or systemic steroids in the six weeks preceding study, since this would likely affect levels of inflammatory cells and mediators in sputum and plasma [18].

## Conclusion

Based on clinical diagnoses, patterns of inflammatory markers in blood plasma and sputum were discernable in clinical diagnosis groups of respiratory disease in smokers with airway complaints. Multivariately, a smaller set of variables remained significantly associated, which opens the possibility to test predictive values of these variables in larger populations. COPD (GOLD 2) and so-called 'healthy smokers' showed consistent opposite associations with plasma LBP, while chronic bronchitics (GOLD 0) showed relatively predominant lymphocytic inflammation compared to other diagnosis groups. Eosinophilia remained significantly associated with reversibility across the spectrum of respiratory disease in smokers with airway complaints, while other clinical characteristics were not.

## Authors' contributions

NC conceived of the study, participated in the design and statistical analysis and drafted the manuscript. JV carried out the sampling and measurement of mediators and helped to draft the manuscript. TS participated in the design and statistical analysis. JJ and MD participated in the sampling and measurement of mediators. CW and OS participated in its design and coordination. EW conceived of the study, participated in its design and coordination, and helped to draft the manuscript. All authors read and approved the final manuscript.

## Declaration of interest

The author(s) declare that they have no competing interests.

## Pre-publication history

The pre-publication history for this paper can be accessed here:



## Figures and Tables

**Table 1:** Population characteristics. Figures are means (SD).

	COPD n = 17	Asthma n = 11	CB n = 14	HS n = 17
Age (range)	61.4 (41–74)	57.6 (36–73)	53.9 (34–72)	54.6 (42–64)
Gender (f/m)	2/15*	3/8	5/9	11/6*
Smoking status (former/current)	6/11	5/6	4/10	7/10
Packyears	48.4* (29)	23.1* (20)	33.5 (18)	32.3 (13)
FEV_1_/FVC postBD	0.56 (0.13)*	0.70 (0.17)	0.73 (0.2)	0.77 (0.22)*
FEV_1 _postBD (%pred)	59.0* (17)	82.1 (17)	100.7* (17)	103.9* (19)
BD-Reversibility (Δ FEV_1 _%pred)	3.5 (9)	12.1* (7)	4.5 (5)	4.9 (4)

*:p < 0.05 using ANOVA analysis.

CB = chronic bronchitis; HS = healthy smokers

**Table 2:** Patterns of inflammatory markers within clinical diagnosis groups. Results are expressed in means (SD).

Compartment	Inflammatory markers	COPD	Asthma	CB	HS
Sputum cell counts	Volume (ml)	6.7 (0.86)	6.9 (0.95)	6.8 (0.93)	3.4 (0.42)
	Total counts (10^6 ^cells/ml)	4.3 (0.80)	2.3 (0.91)	2.0 (0.5)	4.0 (0.99)
	Neutrophils (%)	81.3* (12.2)	49.0* (25.2)	56.6 (23.2)	62.4 (16.5)
	Eosinophils (%)	1.8 (3.7)	20.2* (30.0)	1.3 (1.5)	0.8 (1.1)
	Lymphocytes (%)	0.7 (1.2)	1.8 (2.7)	3.4* (3.4)	0.9 (0.8)
	Macrophages (%)	16.8* (10.5)	29.1 (24.0)	38.6 (21.9)	35.9 (15.6)

Sputum mediators	sTNF-R55 (pg/ml)	658.2* (785.0)	64.4 (14.6)	97.2 (72.7)	169.2 (135.5)
	sTNF R-75 (pg/ml)	222.7* (289.3)	64.3 (53.9)	87.6 (68.1)	84.6 (68.8)
	LBP (ng/ml)	10.1 (11.2)	9.3 (6.3)	7.9 (8.3)	5.3 (5.7)
	IL-8 (ng/ml)	5.5* (3.9)	2.6 (3.2)	2.7 (1.7)	2.4 (1.5)

Plasma mediators	sTNF-R55 (ng/ml)	2.0* (0.5)	1.6 (0.4)	1.5* (0.3)	1.8 (0.4)
	sTNF-R75 (ng/ml)	2.0* (0.4)	1.7 (0.5)	1.4* (0.3)	1.5 (0.4)
	LBP (μg/ml)	28.2* (11.2)	20.0 (4.7)	22.8 (6.5)	17.6* (4.5)
	IL-8 (pg/ml)	7.7* (1.3)	7.1 (0.3)	7.2 (0.6)	7 (0.0)

(*indicates statistical difference from total, p < 0.05, using univariate regression analysis) CB = chronic bronchitis; HS = healthy smokers

**Table 3:** Univariate and multivariate relations between inflammatory markers in sputum and plasma and clinical diagnosis groups.

Compartment	Inflammatory markers	COPD	Asthma	CB	HS
Sputum cell counts	Neutrophils (%)	+	-		
	Eosinophils (%)		+/++		
	Lymphocytes (%)			+/++	
	Macrophages (%)	-			

Sputum mediators	sTNF-R55 (pg/ml)	+/++			
	sTNF-R75 (pg/ml)	+			
	LBP (ng/ml)				
	IL-8 (ng/ml)	+			

Plasma mediators	sTNF-R55 (ng/ml)	+		-	
	sTNF-R75 (ng/ml)	+		-	
	LBP (μg/ml)	+/++			-/–
	IL-8 (pg/ml)	+			

(+ indicates positive univariate significance; - indicates significant negative univariate relation; ++ indicates positive multivariate significance; -- indicates negative multivariate significance) CB = chronic bronchitis; HS = healthy smokers
